# Quack Advertisements in the Religious Press

**Published:** 1888-07

**Authors:** 


### *QUACK ADVERTISEMENTS IN THE RELIGIOUS PRESS.*

Ever since the time when King Asa was "sick of feet until his disease was exceedingly great, yet in his disease he sought not to the Lord but to the physicians," the clergy (except when sick) seem to have had a strong feeling against the regular practice of medicine. If any person wishes to boom a new patent disease eradicator, he immediately obtains the recommendations and endorsements of a few dozen country clergymen, who, for a small consideration, would recommend hell-fire itself as the best of blood purifiers.

Some forms of religion are of so unhealthy a type, that they render the mind susceptible to all kinds of humbugging. Venders of proprietary remedies recognize this fact, and, as a consequence, prefer the religious press as the best medium for the advertisement and sale of their wares. One devout editor publishes an advertisement recommending "Tansy, etc., pills, the ladies' favorite, warranted to remove all irregularities and *obstructions*," and many others of a similar character are found, which would be suppressed by the editor of any respectable secular journal.

The State Medical Society of Arkansas, recognizing the evils attending the present practices of religious journals, at their last meeting, passed the following resolutions:

*Resolved*, That the members of the State Medical Society of Arkansas have for years observed with pain and mortification the patronage given to charlatanism, in all its multifarious aspects, by the religious press of our country.

*Resolved, further and most specifically*, That the appearance in religious papers, ostensibly published for the inculcation of truth and morality, of serious homilies on prayer and praise, side by side with cures for consumption, cancer, Bright's disease, and other incurable ailments, to which an editorial endorsement is often given, as well as secret preparations under the cloak of remedies for disease, but really intended for purposes of fœticide and other immoral uses, largely tends to shake the confidence of the profession of medicine in the integrity and purpose of the managers and editors of such journals.

*Resolved, further*, That it has been the well-known custom of the profession to render services gratuitously to clergymen, which we do not regret, nor do we propose to recall, yet we must assert that the frequent occurrence of endorsements and recommendations of the clergy of peripatetic doctors and advertising charlatans has, in many instances, been the only reward of our gratuitous services.

*Resolved, further*, That we are aware that the editors of religious newspapers admit the painful situation in which these advertisements place them, and excuse themselves by saying that it is necessary to take these advertisements in order to obtain means to conduct their papers; but, in the language of the orthodox theology, we would say: "Put behind you that damnable doctrine that we must do evil that good may come."

*Resolved, further*, That, as a society, we declare that the continued perpetration of the above offenses by some of the clergy and religious press brings harm to the bodies of their constituency, and damages materially their influence upon the thinking class of the medical profession.

*Resolved*, That the secretary be instructed to furnish copies of these resolutions to the religious and medical press of the United States, to the American Medical Association, and to the state medical societies, soliciting their co-operation in bringing about a correction of these grievous and palpable evils.

Very respectfully,

L. P. GIBSON, M. D.,
*Secretary*.

---

WE call the attention of our readers to the new advertisement of Parke, Davis & Co. in this number of the JOURNAL.

WE have received the following interesting epistle from a Kentucky medical man, who seems to have a severe attack of soreheadedness:

LOUISVILLE, June 17, 1888.

The enclosed clipping* is from the Louisville *Courier-Journal* of two days ago. Its appearance in the secular press caused no wonder, but the wonder was how the good editor of the Buffalo paper, from which it appears to be taken, happened to be taken in by Dr. Windy, and where he, the said Windy, became "conspicuous for scientific work." Now, Mr. Editor, this is news to us. If the said Windy is conspicuous for anything but lack of sense, superabundance of gas and large pretentions, it is not known to the average Kentucky doctor. It is well known that there is a coterie of some six or eight men—men of no professional character at home—who attend medical meetings to blow the bazoo for each other in their hustle for official station, and it is an outrage against the profession and an injury to the medical associations that they so often succeed in the accomplishment of their purpose. Most of these men have not a standing at home that should entitle them to any preferment, and the doctor who wears the *nom de plume* of Windy is conspicuous for lack of merit and professional standing. Your notice was undeserved, and was used as an advertisement in a secular paper. This should be enough to open your eyes as to the real character of the man you gave prominence to. The impudence of the small-fry combination alluded to was most conspicuous at the recent meeting of the American Medical, and caused many of us to blush for our State.

If the American Medical proposes to officer the body and sections with such shysters as referred to, the sooner it ceases to exist, the better not only for the profession, but also for the community. MEDICINE.

---

MESSRS. SCHIEFFELIN & Co. have forwarded to us a specimen of the new antipyretic phenacetine (para-acetphenetedine), for which it is claimed that it compares favorably in its effect with any of the modern antipyretics, while it is free from their deleterious influences. It is analogous with antifebrine in its chemical composition, is an inodorless and tasteless powder, hardly soluble in water, a little more soluble in glycerine, but easily soluble in hot alcohol. The dose is from eight to twelve grains. This new remedy furnishes another valuable agent in the treatment of pyrexia. Its special advantages over antipyrine and antifebrine are not definitely defined as yet, and its field of usefulness will be determined by the profession to whom it is commended by high authority.

---

* The following is from the Buffalo *Medical and Surgical Journal*, one of the oldest and best medical journals in the country: "In electing Dr. Wm. H. Wathen, of Louisville, Ky., to be Chairman of the section on Obstetrics and Diseases of Women, and Dr. N. P. Dandridge, of Cincinnati, O., to be Chairman of the section on Surgery and Anatomy, the American Medical Association has performed graceful acts of recognition of men who have been conspicuous for the scientific work they have accomplished in their respective sections. When a medical society brings such men to the front, it does itself honor."

The American Rhinological Association held its sixth annual meeting at Cincinnati, Ohio, September 12, 13 and 14, 1888.

